# Structural connectivity in recovery after coma: Connectome atlas approach

**DOI:** 10.1016/j.nicl.2023.103358

**Published:** 2023-02-24

**Authors:** Polona Pozeg, Yasser Alemán-Goméz, Jane Jöhr, Dafin Muresanu, Alessandro Pincherle, Philippe Ryvlin, Patric Hagmann, Karin Diserens, Vincent Dunet

**Affiliations:** a Department of Radiology, Lausanne University Hospital and University of Lausanne (CHUV-UNIL), Lausanne 1011, Switzerland; b Connectomics Lab, Department of Radiology, Lausanne University Hospital and University of Lausanne (CHUV-UNIL), Lausanne 1011, Switzerland; c Neurology and Acute Neurorehabilitation Unit, Department of Clinical Neurosciences, Lausanne University Hospital and University of Lausanne (CHUV-UNIL), Lausanne 1011, Switzerland; d Department of Neuroscience, Luliu Hatieganu University of Medicine and Pharmacy, Cluj-Napoca 400347, Romania; e Neurology Unit, Department of Medicine, Hôpitaux Robert Schuman, Luxembourg 2540, Luxembourg; f Laboratory of Cortical Excitability and Arousal Disorders, Department of Clinical Neurosciences, Lausanne University Hospital and University of Lausanne (CHUV-UNIL), Lausanne 1011, Switzerland

**Keywords:** VS, vegetative state, UWS, unresponsive wakefulness syndrome, MCS, minimal conscious state, DWI, diffusion weighted imaging, FA, fractional anisotropy, CRS-R, Coma Recovery Scale revised, DRS, Disability rating scale, MBT-r, Motor Behavior Tool – revised, MNI, Montreal neurological institute, NBS, Network Based Statistics, Structural connectivity, Fractional anisotropy, Disorders of consciousness, Connectome, Coma, Diffusion weighted imaging

## Abstract

**Aim:**

Pathological states of recovery after coma as a result of a severe brain injury are marked with changes in structural connectivity of the brain. This study aimed to identify a topological correlation between white matter integrity and the level of functional and cognitive impairment in patients recovering after coma.

**Methods:**

Structural connectomes were computed based on fractional anisotropy maps from 40 patients using a probabilistic human connectome atlas. We used a network based statistics approach to identify potential brain networks associated with a more favorable outcome, assessed with clinical neurobehavioral scores at the patient’s discharge from the acute neurorehabilitation unit.

**Results:**

We identified a subnetwork whose strength of connectivity correlated with a more favorable outcome as measured with the Disability Rating Scale (network based statistics: *t* >*3.5*, *P* =*.010*). The subnetwork predominated in the left hemisphere and included the thalamic nuclei, putamen, precentral and postcentral gyri, and medial parietal regions. Spearman correlation between the mean fractional anisotropy value of the subnetwork and the score was *ρ* = *−0.60* (*P* <.*0001*). A less extensive overlapping subnetwork correlated with the Coma Recovery Scale Revised score, consisting mostly of the left hemisphere connectivity between the thalamic nuclei and pre- and post-central gyri (network based statistics: *t* >*3.5*, *P* =.*033*; *Spearman’s ρ* *= 0.58, P <.0001*).

**Conclusion:**

The present findings suggest an important role of structural connectivity between the thalamus, putamen and somatomotor cortex in the recovery from coma as evaluated with neurobehavioral scores. These structures are part of the motor circuit involved in the generation and modulation of voluntary movement, as well as the forebrain mesocircuit supposedly underlying the maintenance of consciousness. As behavioural assessment of consciousness depends heavily on the signs of voluntary motor behaviour, further work will elucidate whether the identified subnetwork reflects the structural architecture underlying the recovery of consciousness or rather the ability to communicate its content.

## Highlights

• Coma induced by brain injury is characterized with reduced white matter integrity.
• Structural connectivity can be assessed using human probabilistic connectome atlas.
• Identifying brain structural networks associated with recovery of consciousness.
• Recovery correlates with the thalamus–putamen-sensorimotor cortex connectivity.

## 1 Introduction

Severe brain injury often results in coma (Dikmen et al., 2003), a state characterized by the absence of wakefulness, awareness of one’s self or environment, and lack of any voluntary motor behaviour. Recovery from coma occurs in progressive transition through pathological states, where a patient might gradually regain wakefulness and awareness, the two main components of consciousness (Posner et al., 2017). First, patients may transit from coma to the vegetative state (VS) or unresponsive wakefulness syndrome (UWS) when they recover eye opening and sleep-wake cycles, yet they lack awareness of themselves or the environment. (Monti et al., 2010) Second, when inconsistent but apparent signs of conscious behaviour are observed, e.g. visual fixation or object pursuit, localization of noxious stimulus, or simple command following, patients are instead diagnosed with minimally conscious state (MCS). (Giacino et al., 2002) finally, patients emerge from the MCS once they can functionally communicate, or use objects (Giacino et al., 2002). Although bedside clinical diagnostic is the current standard to evaluate the level of recovery after coma, it is often fallible due to patient’s fluctuating arousal, accompanying lesions in the sensory or motor pathways, or other confounding clinical deficits that limit patient’s ability to interact. (Schnakers et al., 2009, Pincherle et al., 2021, Seel et al., 2010) A high proportion of patients is still being behaviourally misdiagnosed as coma or VS/UWS whereas they may succeed to modulate brain activity during an active fMRI or EEG paradigm. (Owen et al., 2006) These patients, despite the absence of any voluntary motor behaviour, demonstrate the ability of command following behaviour and are considered as patients with cognitive motor dissociation (CMD) instead of disorders of consciousness. (Schiff, 2015, Edlow et al., 2017, Owen, 2015) Next to classical neurobehavioral assessment, quantitative measures like neuroimaging and neurophysiology should therefore be used to improve diagnosis, predict the recovery after coma, and thus optimize patient’s care and treatment management. (Porcaro et al., 2022) This is, however, still hindered by our limited understanding of the neural substrates and mechanisms of recovery from coma.

It has been now widely recognized that disorders of consciousness are disorders of brain connectivity (Laureys and Schiff, 2012), affecting in particular thalamo-cortical and fronto-parietal connections. This has been shown by a growing body of studies on functional connectivity, reporting reduced thalamo-cortical connectivity (Sontheimer et al., 2021, Monti et al., 2015) and reduced connectivity within and between the brain’s intrinsic networks (Vanhaudenhuyse et al., 2010, Boly et al., 2009, Silva et al., 2015, Qin et al., 2015, Bodien et al., 2017, Demertzi et al., 2015, Song et al., 2018, Wu et al., 2015) in patients in pathological recovery after coma.

In contrast to the large body of research on functional connectivity, much less is known about the structural connectivity alterations and their association with the recovery after coma. Structural connectivity is defined as the existence of white matter tracts physically interconnecting brain regions and can be evaluated with diffusion weighted imaging (DWI) by recording an MRI sequence measuring differences in local movement of water molecules throughout the brain tissue. The DWI takes the advantage of the diffusion properties of different tissues and can assess impairments of white matter architecture in different pathological conditions. (Hagmann et al., 2006, Soares et al., 2013) DWI studies showed that global white matter integrity, inferred from the DWI-derived metric of fractional anisotropy (FA), reduces with increased impairment of consciousness. (Bodart et al., 2018, Zhang et al., 2017) Tissue structural differences between VS/UWS and MCS patients were found in the subcortical regions, thalamic nuclei, (Fernández-Espejo et al., 2011, Xu et al., 2017) and the cingulate cortex. (Zhang et al., 2017).

With the advancement of neuroimaging and computational techniques, it has become possible to model brain nerve fibers with tractography and construct connectomes, i.e. quantitative representations of brain network connectivity. (Jeurissen et al., 2019) such connectomes allow quantitative analyses of the strength of connectivity between brain regions and its association with clinical variables. (Moody et al., 2021, Hagmann et al., 2008) Brain tractography studies in patients with disorders of consciousness demonstrated notably reduced connectivity between the thalamus, basal ganglia, frontal and parietal cortex. (Weng et al., 2017, Annen et al., 2016, Tan et al., 2019, Yao et al., 2015, Yu et al., 2021).

While the above advancements in neuroimaging enable the non-invasive visualization of brain tracts, severe and widespread brain injuries and deformations may limit the accuracy of brain tractography, (Yeh et al., 2021, Ciccarelli et al., 2008) and affect image spatial normalization and segmentation needed for subsequent group analyses. (Ledig et al., 2015) as fiber tracking is sensitive to the acquisition parameters, it also restricts generalization of findings across subjects with different scanning protocols. (Calabrese et al., 2014).

In the present study, we aimed to identify a subnetwork of structural connectivity associated with functional and cognitive recovery from coma, as evaluated with continuous clinical variables instead of outcome group classifications. To this purpose, we evaluated the topological correlation between clinical scores and white matter integrity using a human white matter connectome atlas (Alemán-Gómez et al., 2022) based approach to circumvent the limitations of tractography when used on severely injured brain images and thereby reduce the associated inter-subject and inter-scanner variability.

## 2 Materials and methods

### 2.1 Patients

This retrospective study was conducted in compliance with the ethical standards of the Declaration of Helsinki and was approved by the local ethical committee (CER-VD, reference number: 142/09). Informed consents to use the patients’ data for research purposes were obtained from patients’ legal representatives. We screened the hospital’s database for adult patients (minimum age of 16 years) who were admitted to the acute neurorehabilitation unit between 1.11.2011 and 31.12.2019, and have suffered from a severe brain injury initially resulting in coma. They were diagnosed with disorders of consciousness based on the Coma Recovery Scale – Revised (CRS-R) (Giacino et al., 2004) criteria at the admission to the unit and had undergone DWI imaging. Patients with low quality imaging data (eddy currents, large susceptibility and motion artefacts) were excluded from the analyses. The image quality was first assessed visually by an experienced radiologist, and then with automatic quality control, using EDDY QC, (Bastiani et al., 2019) where the average absolute motion, average relative motion, and the total outliers percentage were used as the quantitative quality control metrics. Images that exceeded the suggested thresholds (average absolute motion ≥2 mm, average relative motion ≥0.5 mm, total outliers percentage ≥2%) (RPubs, 2023) of two or more metrics were excluded from further analyses.

### 2.2 Clinical scores

As a part of routine clinical evaluation, patients were repeatedly evaluated with various neurobehavioural tests during their stay in the acute neurorehabilitation unit. In our analyses we used the total CRS-R score (0 = absence of any response, 23 = cognitively mediated behaviors) and the Disability rating scale (DRS) (Williams and Smith, 2017) scores (0 = no disability, 29 = extreme vegetative state) at discharge. The items in this scale correspond to the three original World Health Organization categories of impairment, disability, and handicap, and track a patient's functional and cognitive progress from coma to the community. In addition, the patients were also assessed with the Motor Behavior Tool – revised (MBT-r) (Jöhr et al., 2020, Pincherle et al., 2019), a clinical evaluation tool for detecting subtle motor behavior that might reflect residual cognition in unresponsive patients. The patients with detected signs of motor behaviour are identified as patients with clinical cognitive motor dissociation. Experienced clinicians or neuropsychologists carried out the neurobehavioral evaluations. Patients’ demographic and clinical data are presented in Table 1.

**Table 1:** Demographic and clinical data.

Subject	Sex	Age (years)	Interval Injury to MRI (days)	Interval MRI to discharge (days)	Etiology	CRS-R intitial	DRS at disch. (days)	CRS-R at disch. (days)
1	f	67	14	34	CVA	VS/UWS	5	23
2	m	24	8	45	CVA	VS/UWS	22	13
3	m	64	45	28	CVA	VS/UWS	22	4
4	f	57	9	11	CVA	MCS	17	15
5	f	72	101	28	CVA	MCS	25	5
6	m	73	16	41	CVA	VS/UWS	19	16
7	f	67	4	20	TBI	VS/UWS	9	23
8	m	37	1	42	ANOX	COMA	27	4
9	f	35	33	33	TBI	VS/UWS	21	7
10	m	60	21	10	TBI	VS/UWS	9	23
11	m	63	19	63	CVA	MCS	4	22
12	m	55	19	41	CVA	MCS	15	11
13	m	42	31	14	TBI	COMA	15	20
14	f	65	43	8	ANOX	COMA	7	23
15	m	27	287	16	TBI	VS/UWS	20	11
16	m	28	42	8	TBI	MCS	2	23
17	f	37	30	60	TBI	COMA	23	9
18	m	47	16	44	TBI	VS/UWS	7	22
19	f	66	30	28	TBI	COMA	11	23
20	f	39	34	20	CVA	COMA	11	21
21	f	52	23	27	CVA	MCS	15	21
22	m	61	35	27	CVA	COMA	14	18
23	m	61	34	34	CVA	COMA	11	23
24	m	78	50	41	ENC	COMA	15	13
25	m	44	28	26	TBI	COMA	11	21
26	f	60	26	49	CVA	COMA	22	11
27	f	69	41	59	ENC	MCS	18	11
28	f	54	30	25	TBI	VS/UWS	26	5
29	m	50	9	63	ANOX	MCS	8	22
30	f	84	26	14	TBI	COMA	21	13
31	m	16	20	12	TBI	MCS	6	23
32	m	35	19	25	LEUCO	VS/UWS	18	13
33	m	49	29	15	CVA	MCS-	6	21
34	m	72	22	13	CVA	MCS-	7	22
35	m	55	41	47	CVA	COMA	29	22
36	f	58	63	−5	CVA	VS/UWS	9	20
37	f	25	38	7	TBI	VS/UWS	11	11
38	m	73	20	37	TBI	VS/UWS	7	22
39	m	60	38	20	CVA	VS/UWS	11	22
40	m	59	43	3	ANOX	VS/UWS	23	8

CVA = cardiovascular accident, TBI = traumatic brain injury, ANOX = anoxia, ENC = encephalopathy, LEUCO = leucoencephalopathy, VS/UWS = vegetative state or unresponsive wakefulness syndrome, MCS = minimally conscious state, DRS = Disability Rating Scale, CRS-R = Coma Recovery Scale – Revised.

### 2.3 Image acquisition

As this is a retrospective study, the MRI acquisition parameters varied from subject to subject. As such, the 2D spin-echo based DWIs were acquired on different Siemens scanners (Siemens Healthcare, Erlangen, Germany) using various scanning protocols. Four patients were scanned using 1.5 T Aera, two patients using 3 T Skyra, 10 patients using 3 T Verio, and 24 patients using 3 T Prisma Fit scanner. The parameters of the scanning protocol varied from patient to patient: 1.6–3.3 mm slice thickness and interslice gap, 3900–9500 ms TR, 56–100 ms TE, 18–30 diffusion gradient directions at b = 1000 s/mm^2^ and 1–10 gradients at b = 0 s/mm^2.^ More detailed protocol description is given in the Table S2 in the Supplementary material.

### 2.4 Image processing

A DWI preprocessing pipeline was performed using the Mrtrix workflow (Tournier et al., 2019): the images, in native space, were denoised, preprocessed with EDDY (Andersson and Sotiropoulos, 2016) and bias field-corrected. We computed the FA images by fitting a second order tensor model at each voxel using dwi2tensor, and estimating the voxel-vise FA map employing the tensor2metric command. These native and individual FA images were linearly co-registered with their respective-native anatomical image, using FLIRT. (Jenkinson et al., 2002) The Advanced Normalization Tools (ANTs) (Tools, 2022) registration suite was used to non-linearly transform the native anatomical images into the Montreal Neurological Institute (MNI: ICBM 2009c Nonlinear Asymmetric 1x1x1 mm standard space) (Fonov et al., 2011) stereotactic space using *antsRegistrationSyN.sh**,* (Avants et al., 2011) and the derived transformation matrices were subsequently applied to the individual FA image. The alignment of the FA images to the MNI template was verified for each subject and if necessary, manually corrected using the ITK-SNAP software. (Yushkevich et al., 2006).

### 2.5 Atlas-based connectivity matrix

Structural connectomes were calculated on the basis of the multi-scale probabilistic atlas of human connectome. (Alemán-Gómez et al., 2022) This atlas was derived from the diffusion data of 66 healthy adult subjects included in the Human Connectome Project. It models white matter connectivity between cortical and subcortical grey matter regions, parcellated at 4 different scales (Lausanne 2018 parcellation). (Alemán-Gómez et al., 2022) Each normalized FA image was overlaid with the probabilistic tractography atlas and the mean FA values were calculated for each bundle connecting each pair of regions of the scale 1 (95×95 regions). The connectivity strength in the structural connectome thus presented the mean FA values along the voxels belonging to the bundle connecting each pair of regions within the selected parcellation scheme. To exclude voxels with low probability from the selected bundle, the calculations were limited only to the connections present in 80% of the population, and to the voxels belonging to the bundle in 90% of the subjects. (Alemán-Gómez et al., 2022).

### 2.6 Network based statistics (NBS)

Network Based Statistics (NBS) (Zalesky et al., 2010) was used to assess the correlation between the strength of structural connectivity with the clinical scores at discharge. NBS is a validated nonparametric statistical method to evaluate group differences or relationships between variables in large networks, whilst dealing with multiple comparisons problem. It first univariately tests every connection within the matrix, and then identifies any connected structures (components) above the specified test-statistic threshold. The p-values are then assigned to suprathreshold components by indexing their size with the null distribution of maximal component size through permutation testing, controlling for the family-wise error rate. (Zalesky et al., 2010).

Using the NBS toolbox, (NITRC: Network-Based Statistic (NBS) (2022)) we conducted a linear regression between the structural connectivity presented as mean FA values in the connectomes and the two clinical scores at discharge (DRS and CRS-R) while controlling for the patient’s age, sex, and scanning acquisition parameters (the number of diffusion gradient directions, echo time, repetition time, and interslice gap). As the acquisition parameters showed high inter-dependence, we reduced the multicollinearity by aggregating them into one variable using principal component analysis. The scores derived from the loadings of the first component were then used as a single nuisance covariate in the NBS analysis. A detailed description of this step is given in the Supplementary material.

Each subject’s matrix consisted of 95×95 nodes presenting the cortical and subcortical regions as defined by the Lausanne 2018 atlas, (Cammoun et al., 2012) and each element of the matrix (edge) presented the connectivity strength of the respective nodal pair. Statistical significance of correlation between the edges and the clinical score at discharge was assessed through *t*-test for correlation coefficient, by specifying the corresponding contrast, with 10.000 permutations, at the *P-value* of *0.05* (as defined with the NBS method). We evaluated the presence of significant correlations at the primary *t-value* threshold of *3.5*, which corresponded to *P =.001* at 39 degrees of freedom. As the test-statistic threshold influences the extent of the returned subnetwork, and its value has not been standardly defined, it has been suggested to assess the extent of subnetwork using different thresholds. (Zalesky et al., 2010) Therefore, in addition to the primary threshold of *t = 3.5*, we estimated the extent of the significant network by increasing and decreasing this threshold.

## 3 Results

### 3.1 Demographic and clinical scores

We initially identified 148 patients in the hospital’s database who were diagnosed with disorders of consciousness based on the CRS-R criteria. The DWI images acquired after the brain insult resulting in the disorders of consciousness diagnosis were available for 52 patients. Their age ranged between 16 and 83 years (mean = 51.7, SD = 18.3). The data of 7 patients were first removed due to the presence of larger magnetic susceptibility artefacts, and 2 patients were excluded due to the DWI interslice inconsistencies. 4 more patients were additionally excluded due to larger motion artefacts as assessed by the automatic quality control. The final sample thus consisted of 40 patients (16 females, 16–84 years, mean = 53.5, SD = 16.4; Kolmogorov-Smirnoff test of normality: *D* = *0.11*, *P* =.*65*).

According to the initial evaluation with the CRS-R at the admission to the acute neurorehabilitation unit, 13 patients were diagnosed with coma, 16 with UWS, and 11 with MCS. Using recently developed Motor Behavior Tool revised (MBT-r) (Jöhr et al., 2020, Pincherle et al., 2019), residual consciousness was detected in 31 patients, and they were identified as having a clinical cognitive motor dissociation, whereas no signs of subtle motor behaviour were observed in 9 patients, who were consequently identified as having a true disorder of consciousness. Patients’ MBT-r evaluation is presented in the Supplementary Table 1.

Median time interval between the injury and MRI scan was 29.5 days, (*IQR = 20.5*, Kolmogorov-Smirnoff test of normality: *D = 0.32, P <.001*) and the median time interval between the MRI scan and discharge was 27 days (*IQR = 27*; Kolmogorov-Smirnoff test of normality: *D = 0.11, P =.70*).

At the discharge from the unit, the median DRS score was 14.5 (*IQR = 12.5,* Kolmogorov-Smirnoff test of normality*: D = 0.16, P =.22*) and the median total CRS-R score was 20 (*IQR = 11*, Kolmogorov-Smirnoff test of normality: *D = 0.23, P =.02*). Brain injury etiologies included traumatic brain injuries (*n = 15*), cerebrovascular accident (*n = 18*), anoxia (*n = 4*), encephalopathy (*n = 2*), and leucoencephalopathy (*n = 1*). Patients’ demographic and clinical data are presented in Table 1. Based on qualitative lesion evaluation (see Supplementary material) we found no significant differences in the injury severity between the left and right hemisphere (*z* = *−0.39*, *P* =.*70*). We have observed three subjects with midline shift (maximal distance between the midline and the septum pellucidum: 3.5, 6.1 and 7.5 mm, respectively). The midline shift was corrected during the normalization step for all three patients. Lesion location information is described in Supplementary Table 1.

### 3.2 Network based statistics (NBS)

At the primary threshold of *t >3.5*, NBS showed a significant association between the DRS score and the strength of connectivity in a subnetwork comprising 29 nodes and 41 edges at *P =.010*. The greater strength of the connectivity within this subnetwork was found for lower DRS score – i.e.,more favourable clinical outcome at discharge. The majority of connections was located within the left hemisphere and mostly consisted of connectivity between thalamic nuclei, putamen, precentral and postcentral gyrus, and medial parietal regions. The node with the highest degree, i.e. the number of edges, in the subnetwork was the left precentral gyrus (*nodal degree = 16*), followed by the left putamen (*nodal degree = 7*) and the left superior frontal gyrus (*nodal degree = 7*). Spearman’s correlation coefficient between the averaged FA values across the edges in the subnetwork and the DRS score at discharge was *ρ = – 0.60 (P <.0001)*. A sparser subnetwork of 13 nodes and 17 edges was found for a *t-value* at *3.7 (P =.011).* On the other hand, lowering the threshold to *t >3.3* showed a more extensive subnetwork of 38 nodes and 86 edges *(P =.009)*. The length of the interval between the brain injury and the DWI scanning did not significantly correlate with the mean FA values of the primary subnetwork (*Spearman’s ρ* = −*0.05, P =.75).*

A less extensive, but overlapping subnetwork was also found to positively correlate with the CRS-R score at discharge at the *t-value* of *3.5*, demonstrating stronger connectivity for a more favorable recovery at discharge. This subnetwork consisted of 8 nodes and 8 edges *(P =.033),* depicting connectivity between thalamic nuclei and pre-and postcentral gyrus in the left hemisphere. The left precentral gyrus had the highest degree (4) in the subnetwork, followed by the left postcentral gyrus (*nodal degree = 3*). Spearman’s correlation coefficient between the averaged FA values across the edges in the subnetwork and the CRS-R score at discharge was ρ *= 0.58 (P <.0001)*. No significant connections were found for *t >3.7*, whereas lower thresholds of *t >3.3* resulted in a moderate increase in the extent of the subnetworks, consisting of 16 nodes and 22 edges *(P =.026)*. The length of time elapsed between the brain injury and the day of DWI scanning did not significantly correlate within the mean FA values of the subnetwork (*Spearman’s ρ* = −*0.13, P =.41*).

The main effect of patient’s sex, age, or acquisition parameters did not result in any significant structural connectivity subnetwork (NBS analysis: 2-tailed *t*-test at *t >3.5*, 10,000 permutations, all *p* *>*.*05*).

**Fig. 1:**
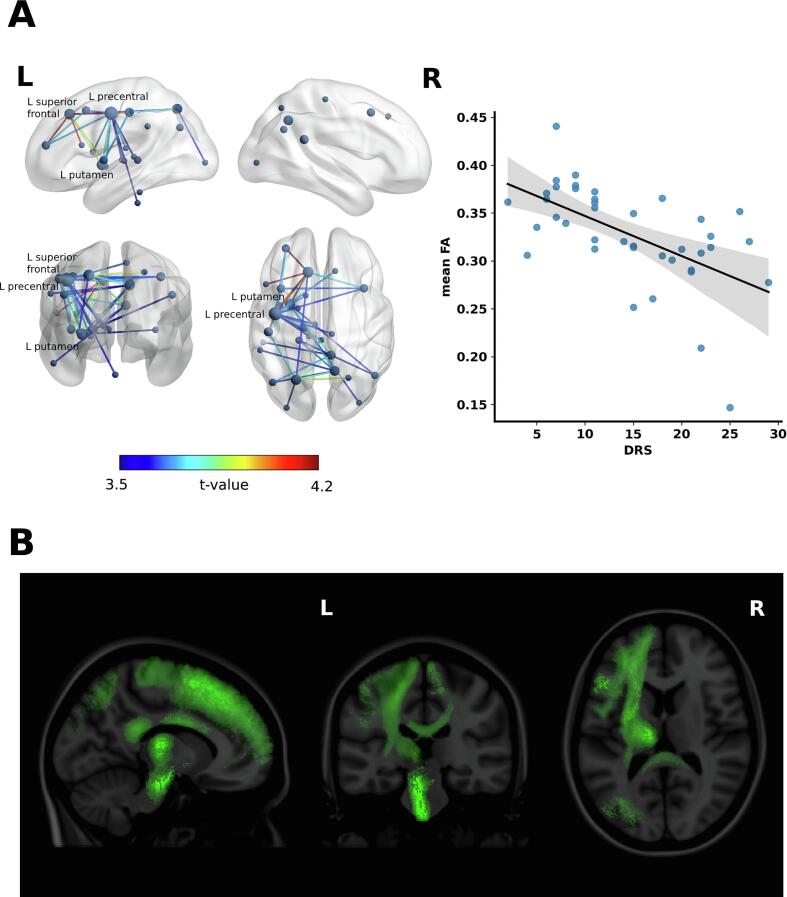
**Subnetwork correlating with the Disability Rating Scale (DRS) score. (A)** Left: The 3D view of the structural connectivity subnetwork that significantly correlates with the DRS at the patient’s discharge from the acute neurorehabilitation unit. The subnetwork is displayed at the test-statistic threshold *t > 3.5*. The color of the edges represents the t-value of correlation. The nodal degrees are represented with a relative size of the nodes in the network. The node with the highest degree is the left precentral gyrus (*nodal degree = 16*), followed by the left putamen (*nodal degree = 7*) and the left superior frontal gyrus (*nodal degree = 7*). The brain network is visualized using the BrainNet Viewer. (Xia et al., 2013, NITRC: BrainNet Viewer, 2022) Right: Scatter plot showing correlation between the average fractional anisotropy (FA) value across the connections in the subnetwork and the DRS score (Spearman’s *ρ = −0.60, P <.0001*). The shaded area represents a 95% confidence interval of the fitted line. **(B)** The subnetwork significantly correlating with the DRS score shown as probabilistic white matter fiber bundles of the human connectome atlas. (Alemán-Gómez et al., 2022).

**Fig. 2:**
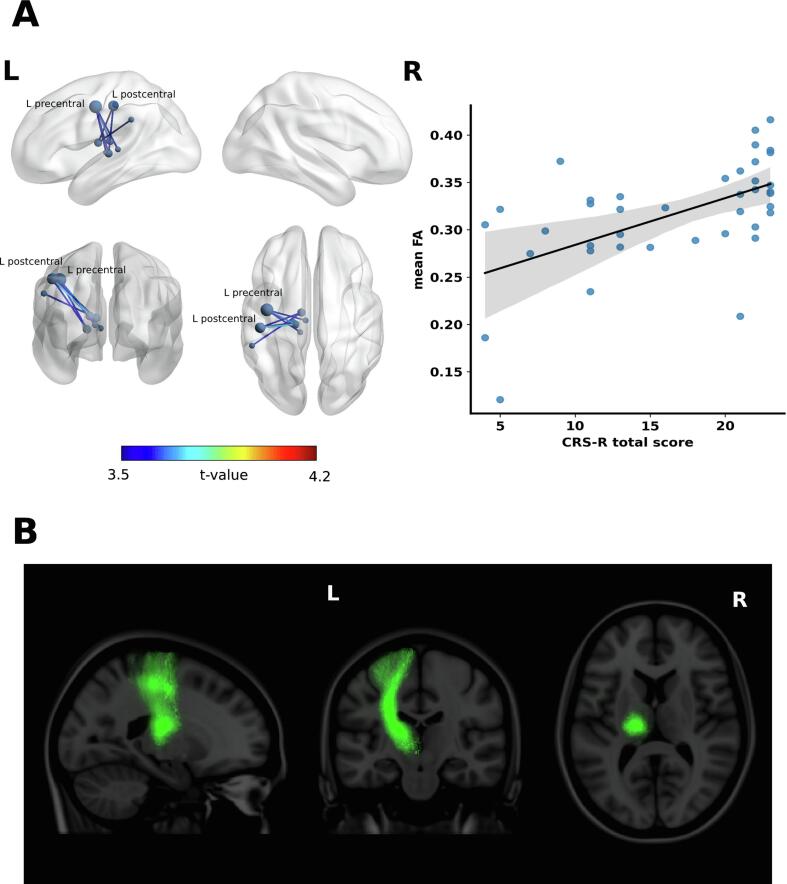
**Subnetwork correlating with the Coma Recovery Scale – revised (CRS-R) score. (A)** Left: The 3D view of the structural connectivity subnetwork that significantly correlates with the CRS-R at the patient’s discharge from the acute neurorehabilitation unit. The subnetwork is displayed at the test-statistic threshold *t > 3.5*. The color of the edges represents the t-value of correlation. The nodal degrees are represented with a relative size of the nodes in the network. The left precentral gyrus has the highest nodal degree (4), followed by the left postcentral gyrus (*nodal degree = 3*). The brain network is visualized using the BrainNet Viewer. (Xia et al., 2013, NITRC: BrainNet Viewer, 2022) Right: Scatter plot showing correlation between the average fractional anisotropy (FA) value across the connections in the subnetwork and the CRS-R score (Spearman’s *ρ = 0.58, P <.0001*). The shaded area represents a 95% confidence interval of the fitted line. **(B)** The subnetwork significantly correlating with the CRS-R score shown as probabilistic white matter fiber bundles of the human connectome atlas. (Alemán-Gómez et al., 2022).

Graphical depictions of the subnetworks that significantly correlate with the DRS and CRS-R score are shown in Fig. 1 and Fig. 2, respectively. The t-values of the connections of each nodal pair in the subnetwork are presented in Table 2.

**Table 2:** Significant nodal pairs at t >3.5 for the correlation with the DRS and CRS-R.

Correlation with clinical score	Atlas region	Atlas region	t-value	
			DRS	CRS-R
DRS	right caudal middle frontal	left superior frontal	3.72	
DRS	left pars opercularis	left superior frontal	4.09	
DRS	left rostral middle frontal	left superior frontal	4.18	
DRS	right caudal middle frontal	left caudal middle frontal	3.63	
DRS	left rostral middle frontal	left caudal middle frontal	3.70	
DRS	right superior frontal	left precentral	3.67	
DRS	right caudal middle frontal	left precentral	3.60	
DRS	right paracentral	left precentral	3.62	
DRS	left rostral middle frontal	left precentral	3.67	
DRS	left superior frontal	left precentral	4.13	
DRS	left caudal middle frontal	left precentral	3.66	
DRS	left precentral	left postcentral	4.04	
DRS	right precuneus	left supramarginal	3.56	
DRS	right isthmus cingulate	left superior parietal	3.84	
DRS	right superior parietal	left superior parietal	3.88	
DRS	right precuneus	left superior parietal	3.77	
DRS	left superior frontal	left superior parietal	3.60	
DRS	left postcentral	left superior parietal	3.73	
DRS	right isthmus cingulate	left inferior parietal	3.69	
DRS	right precuneus	left inferior parietal	3.51	
DRS	left superior parietal	left lateral occipital	3.57	
DRS	right isthmus cingulate	left inferior temporal	3.56	
DRS	left superior frontal	left insula	3.90	
DRS	left precentral	left insula	3.74	
DRS	left postcentral	left insula	3.67	
DRS	right inferior parietal	left thalamus (Pul)	3.53	
DRS	right precuneus	left thalamus (Pul)	3.65	
DRS/CRS-R	left precentral	left thalamus (MD)	3.54	3.53
DRS/CRS-R	left precentral	left thalamus (LP-VP)	3.72	3.68
DRS/CRS-R	left precentral	left thalamus (PuM)	3.53	3.54
DRS/CRS-R	left precentral	left thalamus (VP-VL)	3.58	3.56
DRS	left precentral	left caudate	3.65	
DRS	right isthmus cingulate	left putamen	3.77	
DRS	right inferior parietal	left putamen	3.60	
DRS	right precuneus	left putamen	3.70	
DRS	right lateral occipital	left putamen	3.57	
DRS	left superior frontal	left putamen	3.85	
DRS	left precentral	left putamen	3.78	
DRS	left postcentral	left putamen	3.71	
DRS	left precentral	left pallidum	3.66	
DRS	left precentral	brain stem	3.54	
CRS-R	left postcentral	left thalamus (LP-VP)		3.73
CRS-R	left postcentral	left thalamus (VL)		3.51
CRS-R	left supramarginal	left thalamus (VL)		3.53
CRS-R	left postcentral	left thalamus (VP-VL)		3.55

LP-VP = lateral posterior ventral posterior nucleus, VP-VL = ventral posterior ventrolateral nucleus, MD = mediodorsal nucleus, Pul = pulvinar, PuM = medial pulvinar centrolateral nuclei, VL = ventrolateral nucleus.

## 4 Discussion

In this retrospective study, we used an atlas-based approach to investigate the relationship between brain structural connectivity and clinical outcome in the pathological recovery after coma. To this goal, we applied the NBS method to assess the association between white matter integrity measured by FA scalar maps and the level of disability, evaluated with the DSR and CRS-R scales at the patient’s discharge from the acute neurorehabilitation unit. We identified a brain subnetwork that significantly correlated with the DRS score, and mostly consisted of connectivity in the left hemisphere between the thalamic nuclei, putamen, precentral and postcentral gyrus, as well as superior frontal, superior parietal regions, and the brainstem. An overlapping but less extensive subnetwork was also found to correlate with the total CRS-R score, and included mostly the thalamic radiations to the precentral and postcentral gyrus of the left hemisphere.

The present findings ascribe and confirm an important role of the subcortico-cortical structural connectivity in the recovery from coma, elucidating in particular the integrity between somatomotor cortex, putamen, and thalamic nuclei. These structures are considered to have a key role in the motor circuit within the cortico-basal ganglia-thalamo-cortical loop, involved in the generation and modulation of voluntary movement. (Draganski et al., 2008, DeLong and Wichmann, 2007) The identified subnetwork is also part of the negative feedback loop, which, as suggested by the *meso*-circuit hypothesis, is crucial for the maintenance of excitatory outflow to the cortex and therefore sustenance of consciousness. (Laureys and Schiff, 2012, Schiff, 2010) According to the *meso*-circuit hypothesis, interruption of the circuit between the frontal lobe, striatum, pallidum, and central thalamus results in disinhibition of globus pallidus, leading to excessive inhibition of the thalamus and consequently suppression of cortical activity. Supportive evidence for the *meso*-circuit hypothesis comes from recovery of consciousness after deep brain stimulation of the thalamus, (Schiff et al., 2007) transcranial direct stimulation of prefrontal cortex, (Thibaut et al., 2014) or after administration of zolpidem, a hypnotic, decreasing the globus pallidus inhibition of the thalamus. (Cohen and Duong, 2008, Whyte and Myers, 2009) Analogous to our study, and consistent with the *meso*-circuit hypothesis, were findings on structural connectivity reported by Weng et al., (Weng et al., 2017) who showed reduced connectivity of the basal ganglia and thalamus with the frontal cortex in the patients with disorders of consciousness as compared to healthy controls, while (Zheng et al., 2016, Yao et al., 2015, Yu et al., 2021) demonstrated that white matter integrity between thalamus and sensorimotor cortex could distinguish between patients with various degrees of impairment of consciousness. Our results are also consistent with studies that used different imaging modalities, showing that the restoration of functional MRI and metabolic thalamo-cortical connectivity correlated with the recovery of consciousness and complex cognitive behaviour, (Laureys et al., 2000, Crone et al., 2018) or demonstrated that lesions of the basal ganglia, thalamus, and mesencephalon are predictive of an adverse outcome in the disorders of consciousness. (Pozeg et al., 2021, Rohaut et al., 2019).

However, preserved structural integrity of the *meso*-circuit is a necessary, but not a sufficient foundation for awareness. While the thalamus and striatum have a central role in the arousal regulation and modulation of the excitatory input to the cortex, awareness critically depends on adequate cortico-cortical communication, mostly driven by long-range connections between frontal and parietal regions. (Modolo et al., 2020, Noirhomme et al., 2010) numerous studies have indeed shown more preserved cortical connectivity in the MCS than in the VS/UWS patients, (Vanhaudenhuyse et al., 2010, Silva et al., 2015), and that preserved within and between cortical networks connectivity can be predictive of successful recovery from coma. (Bodien et al., 2017) likewise, widespread brain injury affecting larger cortical areas was associated with more adverse outcome after coma. (Pozeg et al., 2021) the structural subnetwork revealed by our analyses included thalamo-cortical connections to the superior frontal and parietal regions, but we did not identify extensive correlations between the clinical scores and direct cortico-cortical connections. This could be attributed to the heterogeneity of our sample in terms of lesion location and distribution, aetiology, and clinical severity. In the presence of such heterogeneity, the current results could possibly reflect the thalamo-cortical connectivity as a common denominator of the white matter integrity necessary (although not sufficient) for the successful recovery after coma in our sample.

The subnetwork identified in our analysis is mainly lateralized to the left hemisphere. This lateralized finding is not due to possibly more frequent and severe right hemispheric lesions, as the lesions were equally distributed across two hemispheres in our sample. Alternatively, the left somatomotor cortex with its connections to the ventrolateral thalamus is specialised for planning and execution of movements, (Gale et al., 2021, Bosch-Bouju et al., 2013) including speech production. (Behroozmand et al., 2015, Simonyan and Fuertinger, 2015) significant correlation between the clinical scores and the white matter integrity of this network might in fact reflect the neural structure underlying the ability to verbally or functionally communicate and interact with the environment. Assessment of consciousness with the standard clinical neurobehavioral tools indeed heavily depends on the detection of signs of voluntary motor behaviour. Reduced connectivity within this network might therefore not indicate a loss of consciousness, but rather mask the ability to communicate its content. In such cases, covert awareness might be detected through functional MRI or electroencephalography paradigms, where the patient demonstrates command following ability through motor imagery tasks, (Owen et al., 2006) and the condition is described as “cognitive motor dissociation” (CMD). (Schiff, 2015, Edlow et al., 2017) exact underlying pathology of the CMD is not yet understood, although recent research has pointed to the possible involvement of lesions to the somatomotor-striato-thalamic network in the CMD patients, blocking the motor output or its initiation, and producing neurological disorders that hinder interaction with the environment, such as akinetic mutism, aphasia, or abulia. (Pincherle et al., 2021, Jöhr et al., 2022, Fernández-Espejo et al., 2015, Ghoshal et al., 2011) our findings are in contrast with the study of Tan et al. (Tan et al., 2022) who reported significant correlation between the CRS-R scores and FA values of the mostly right hemisphere white mater tracts. The authors associated this hemispheric asymmetry with the role of the right hemisphere in the bodily self-awareness and its disorders (Ronchi et al., 2018), although they have not excluded a possible effect of neural compensation after brain injury. The present study cannot disambiguate whether the structural subnetwork we found in our study represents a supporting structural architecture for the recovery of consciousness after coma, or whether it reflects a neural pathway to communicate awareness. This question should be addressed in a future study, which will use multimodal imaging and motor behaviour-independent evaluation of consciousness.

Overall, this study demonstrates that the atlas-based approach presents a feasible and pragmatic method to study structural connectivity in a population with severe brain injuries. Its main advantage is the possibility to create structural connectomes without fiber tracking, contrary to tractography, which is sensitive to diffusion MRI acquisition parameters (Yeh et al., 2021) and has a questionable validity if performed on a brain with larger anomalies. (Calabrese et al., 2014) second, an atlas-based approach to assess structural connectivity is easier to use also for non-expert clinical researchers and it is less demanding with regards to the software equipment and computer processing complexity. Nevertheless, this study has certain important limitations that need to be addressed. First, the accuracy of brain normalization to a template might be compromised in the presence of the severe pathology, (Ledig et al., 2015) and consequently introduce additional bias in the analysis. Using lesion masking during normalization step could potentially reduce such bias, however its normalization improvement is limited in the presence of larger and bilateral lesions. (Brett et al., 2001) second, we have quantified the white matter structural integrity using FA scalar maps, where a high value generally indicates a high microstructural organization of the tissue, and an FA reduction, a possible pathology. However, in the presence of crossing or mixing fibers, the FA is lower, even despite preserved fiber integrity, which can lead to erroneous interpretation of results. (Figley et al., 2022) this drawback can be improved with modern diffusion techniques that are more robust to crossing fibers. (Figley et al., 2022) in addition, a possible confounding effect of edema should also be considered. Increased water content due to edema reduces the FA values even though the axonal fibers might be intact. (Assaf and Pasternak, 2008) we have chosen to perform the current analysis based on the FA values since this scalar has been most commonly used to study structural connectivity, and has also shown to most strongly correlate with the levels of consciousness in traumatic brain injury. (Zhang et al., 2017) additional information about the nature of the brain’s structural changes could be potentially obtained by performing similar network analysis on different DWI scalars, such as mean, axial, and radial diffusivity. Finally, due to the retrospective nature of the study, our results are possibly confounded with the heterogeneity of our sample in terms of age, aetiology, lesion type and locations as well as by the variability of MRI acquisition parameters. For this reason, the present findings should preferably be reproduced with advanced diffusion imaging approaches in a prospective study.

In conclusion, this study demonstrates a significant association between the level of white matter integrity and clinical outcome in patients with pathological recovery after coma. Using a connectome atlas-based approach we identified a structural connectivity subnetwork, which correlated with better recovery after coma, predominantly in the left hemisphere and mainly included connections between thalamus, putamen, precentral and postcentral gyrus. The present findings might advance our current understanding of neural biomarkers for predicting the recovery after coma, and may contribute to the application of neuroimaging in the assessment and rehabilitation planning of patients with severe brain injuries.

## CRediT authorship contribution statement

**Polona Pozeg:** Conceptualization, Methodology, Data curation, Visualization, Writing – original draft, Writing – review & editing. **Yasser Alemán-Goméz:** Methodology, Writing – review & editing. **Jane Jöhr:** Data curation, Writing – review & editing. **Dafin Muresanu:** Writing – review & editing. **Alessandro Pincherle:** Investigation, Writing – review & editing. **Philippe Ryvlin:** Writing – review & editing. **Patric Hagmann:** Conceptualization, Writing – review & editing. **Karin Diserens:** Investigation, Writing – review & editing, Funding acquisition. **Vincent Dunet:** Conceptualization, Methodology, Writing – review & editing, Supervision, Funding acquisition.

## Declaration of Competing Interest

The authors declare that they have no known competing financial interests or personal relationships that could have appeared to influence the work reported in this paper.
