# Supplementary material for: Structural connectivity in recovery after coma: Connectome atlas approach
Source: Neuroimage Clin. 2023 Feb 24;37:103358. doi: 10.1016/j.nicl.2023.103358 (PMC9996111; doi:10.1016/j.nicl.2023.103358)
Supplement: Supplementary data 1 [file mmc1.docx]

# Supplementary material

**Qualitative assessment of lesion extent and laterality**

With the aim to evaluate a possible hemispheric prevalence of brain injuries in our sample we performed a qualitative lesion evaluation^1^. We evaluated the presence of lesions in four peripheral regions (frontal, temporal, parietal, and occipital lobe) and in five deep regions (basal ganglia, thalamus, mesencephalon, pons and cerebellum) for each brain hemisphere, resulting in a total of 18 regions. Based on the severity of the lesion, each region was assigned a score as follows: 0 if the region was intact, 1 for a presence of a small, focal lesion, and 2 for a presence of a diffuse or a large lesion (lesion volume ≥ 30% of the region). Comparing the lesion scores between the left and right hemisphere did not reveal any significant differences (2-tailed Mann-Whitney U test: z = -0.39, P = .70). We further compared the two hemispheres in regards to the severity of injury in the regions implicated in the subnetwork, which was found to significantly correlate with the clinical outcome (frontal lobe, parietal lobe, basal ganglia, and thalamus). We found no significant differences in severity of injury between the left and the right hemisphere subnetwork (2-tailed Mann-Whitney U test: z = -0.70, P = .48).

Lesion count and severity, calculated as a sum of scores across all regions, significantly correlated with the Disability rating score (Spearman’s r = 0.41, P = .006) and marginally with the Coma Recovery scale (Spearman’s r = - 0.31, P = 0.051), indicating that patients with more widespread brain injuries had worse clinical outcomes. Lesion scores are presented in Table S1 in the Supplementary Material.

**Supplementary Table S1**. Identification of residual consciousness and qualitative assessment of lesion location and lesion laterality

| **Sub** | **MBTr** | **Lesion** | **FL** | **FR** | **TL** | **TR** | **PL** | **PR** | **OL** | **OR** | **BGL** | **BGR** | **THL** | **THR** | **MESL** | **MESR** | **PONSL** | **PONSR** | **CERL** | **CERR** |
| --- | --- | --- | --- | --- | --- | --- | --- | --- | --- | --- | --- | --- | --- | --- | --- | --- | --- | --- | --- | --- |
| 1 | cCMD | BL | 1 | 1 | 0 | 0 | 1 | 0 | 0 | 0 | 1 | 0 | 0 | 0 | 0 | 0 | 0 | 0 | 0 | 0 |
| 2 | DOC | BL | 2 | 2 | 0 | 2 | 2 | 2 | 1 | 0 | 2 | 1 | 0 | 0 | 2 | 0 | 2 | 0 | 0 | 0 |
| 3 | cCMD | BL | 0 | 0 | 0 | 0 | 0 | 0 | 0 | 0 | 0 | 1 | 1 | 0 | 0 | 0 | 0 | 0 | 0 | 0 |
| 4 | cCMD | BL | 2 | 1 | 2 | 0 | 2 | 0 | 2 | 0 | 0 | 0 | 0 | 0 | 0 | 0 | 0 | 0 | 0 | 0 |
| 5 | cCMD | BL | 2 | 2 | 2 | 2 | 2 | 2 | 2 | 1 | 2 | 1 | 0 | 0 | 2 | 0 | 0 | 0 | 0 | 0 |
| 6 | cCMD | BL | 1 | 0 | 1 | 0 | 1 | 1 | 1 | 0 | 0 | 0 | 0 | 0 | 0 | 0 | 0 | 0 | 1 | 0 |
| 7 | cCMD | BL | 2 | 2 | 1 | 2 | 1 | 2 | 0 | 0 | 0 | 0 | 0 | 0 | 0 | 0 | 0 | 0 | 1 | 2 |
| 8 | DOC | BL | 2 | 2 | 2 | 2 | 2 | 2 | 2 | 2 | 2 | 2 | 0 | 0 | 0 | 0 | 0 | 0 | 0 | 0 |
| 9 | DOC | BL | 2 | 2 | 2 | 1 | 1 | 0 | 1 | 0 | 0 | 1 | 0 | 0 | 0 | 0 | 0 | 0 | 0 | 1 |
| 10 | cCMD | BL | 2 | 1 | 1 | 1 | 0 | 0 | 0 | 0 | 0 | 0 | 0 | 0 | 0 | 0 | 0 | 0 | 0 | 0 |
| 11 | cCMD | R | 0 | 2 | 0 | 2 | 0 | 0 | 0 | 0 | 0 | 2 | 0 | 2 | 0 | 2 | 0 | 0 | 0 | 0 |
| 12 | cCMD | BL | 2 | 1 | 2 | 2 | 2 | 0 | 0 | 0 | 2 | 2 | 2 | 1 | 1 | 0 | 2 | 2 | 1 | 0 |
| 13 | cCMD | BL | 2 | 2 | 2 | 2 | 2 | 1 | 2 | 2 | 2 | 1 | 0 | 0 | 0 | 0 | 0 | 0 | 0 | 0 |
| 14 | cCMD | BL | 0 | 0 | 0 | 0 | 0 | 0 | 0 | 0 | 0 | 0 | 0 | 0 | 0 | 0 | 0 | 0 | 0 | 0 |
| 15 | DOC | BL | 2 | 2 | 2 | 2 | 1 | 2 | 1 | 2 | 0 | 0 | 0 | 0 | 2 | 2 | 2 | 2 | 2 | 2 |
| 16 | cCMD | BL | 0 | 2 | 2 | 2 | 0 | 1 | 0 | 0 | 0 | 0 | 0 | 0 | 0 | 0 | 0 | 0 | 0 | 0 |
| 17 | DOC | R | 0 | 2 | 0 | 2 | 0 | 2 | 0 | 2 | 0 | 2 | 0 | 2 | 0 | 0 | 0 | 0 | 0 | 0 |
| 18 | cCMD | BL | 2 | 2 | 0 | 2 | 0 | 2 | 0 | 2 | 0 | 0 | 0 | 1 | 2 | 0 | 0 | 0 | 0 | 0 |
| 19 | cCMD | BL | 2 | 2 | 2 | 2 | 0 | 0 | 0 | 0 | 0 | 0 | 0 | 0 | 0 | 0 | 0 | 0 | 0 | 0 |
| 20 | DOC | BL | 2 | 0 | 0 | 0 | 0 | 0 | 0 | 0 | 1 | 2 | 0 | 2 | 0 | 0 | 0 | 0 | 0 | 0 |
| 21 | cCMD | L | 2 | 0 | 2 | 0 | 2 | 0 | 0 | 0 | 2 | 0 | 0 | 0 | 2 | 0 | 0 | 0 | 0 | 0 |
| 22 | cCMD | BL | 1 | 0 | 0 | 0 | 2 | 0 | 0 | 0 | 0 | 0 | 1 | 2 | 0 | 0 | 0 | 0 | 0 | 0 |
| 23 | cCMD | BL | 1 | 2 | 0 | 2 | 0 | 2 | 0 | 2 | 1 | 2 | 0 | 1 | 0 | 2 | 0 | 0 | 0 | 0 |
| 24 | cCMD | R | 0 | 0 | 0 | 0 | 0 | 0 | 0 | 0 | 0 | 0 | 0 | 1 | 0 | 0 | 0 | 0 | 0 | 0 |
| **Sub** | **MBTr** | **Lesion** | **FL** | **FR** | **TL** | **TR** | **PL** | **PR** | **OL** | **OR** | **BGL** | **BGR** | **THL** | **THR** | **MESL** | **MESR** | **PONSL** | **PONSR** | **CERL** | **CERR** |
| 25 | cCMD | BL | 2 | 1 | 2 | 0 | 1 | 0 | 0 | 0 | 0 | 0 | 0 | 0 | 0 | 0 | 0 | 0 | 0 | 0 |
| 26 | cCMD | BL | 2 | 2 | 2 | 0 | 2 | 1 | 0 | 0 | 2 | 0 | 1 | 0 | 1 | 0 | 0 | 0 | 0 | 0 |
| 27 | cCMD | BL | 2 | 2 | 2 | 2 | 2 | 2 | 2 | 2 | 0 | 0 | 2 | 2 | 0 | 0 | 0 | 0 | 1 | 0 |
| 28 | DOC | BL | 0 | 1 | 1 | 1 | 0 | 0 | 0 | 0 | 0 | 0 | 0 | 0 | 2 | 2 | 0 | 0 | 0 | 0 |
| 29 | cCMD | BL | 0 | 0 | 0 | 0 | 0 | 0 | 0 | 0 | 2 | 2 | 0 | 0 | 0 | 0 | 0 | 0 | 0 | 0 |
| 30 | cCMD | BL | 0 | 0 | 1 | 2 | 0 | 0 | 0 | 0 | 0 | 1 | 1 | 1 | 0 | 0 | 0 | 0 | 1 | 2 |
| 31 | cCMD | BL | 2 | 2 | 0 | 2 | 0 | 0 | 0 | 0 | 0 | 2 | 0 | 1 | 0 | 1 | 0 | 0 | 0 | 0 |
| 32 | DOC | BL | 2 | 2 | 0 | 0 | 2 | 2 | 2 | 2 | 0 | 0 | 0 | 0 | 0 | 0 | 0 | 0 | 0 | 0 |
| 33 | cCMD | R | 0 | 2 | 0 | 2 | 0 | 1 | 0 | 0 | 0 | 2 | 0 | 2 | 0 | 2 | 0 | 0 | 0 | 0 |
| 34 | cCMD | BL | 0 | 2 | 0 | 2 | 2 | 2 | 0 | 0 | 0 | 0 | 0 | 2 | 0 | 0 | 0 | 0 | 0 | 0 |
| 35 | cCMD | BL | 2 | 2 | 0 | 0 | 2 | 2 | 0 | 2 | 0 | 0 | 0 | 0 | 0 | 0 | 0 | 1 | 2 | 2 |
| 36 | cCMD | L | 1 | 0 | 0 | 0 | 0 | 0 | 0 | 0 | 0 | 0 | 0 | 0 | 0 | 0 | 0 | 0 | 0 | 0 |
| 37 | cCMD | L | 0 | 0 | 2 | 0 | 0 | 0 | 0 | 0 | 0 | 0 | 0 | 0 | 0 | 0 | 0 | 0 | 0 | 0 |
| 38 | cCMD | BL | 2 | 1 | 2 | 2 | 0 | 0 | 0 | 0 | 0 | 0 | 0 | 0 | 0 | 0 | 0 | 0 | 0 | 0 |
| 39 | cCMD | BL | 2 | 2 | 0 | 0 | 0 | 0 | 0 | 0 | 1 | 1 | 0 | 0 | 0 | 0 | 0 | 0 | 0 | 0 |
| 40 | DOC | BL | 2 | 2 | 2 | 2 | 2 | 2 | 2 | 2 | 2 | 2 | 2 | 2 | 0 | 0 | 0 | 0 | 0 | 0 |

MBTr = Motor behavior Tool revised; cCMD = clinical cognitive motor dissociation; DOC = disorder of consciousness; BL = bilateral; L = left hemisphere lesion; R = right hemisphere lesion; FL = left frontal lobe; FR = right frontal lobe; TL = left temporal lobe; TR = right temporal lobe; PL = left parietal lobe; PR = right parietal lobe; OL = left occipital lobe; OR = right occipital lobe; BGL = left basal ganglia; BGR = right basal ganglia; THL = left thalamus; THR = right thalamus; MESL = left mesencephalus; MESR = right mesencephalus; PONSL = left pons; PONSR = right pons; CERL = left cerebellum; CERR = right cerebellum; 0 = intact region; 1 = focal lesion; 2 = large or diffuse lesion

**Image acquisition**

**Supplementary Table S2**. Detailed MRI acquisition parameters

| N | Scanner | Slice thickness [mm] | Interslice gap  [mm] | TE  [ms] | TR  [ms] | Nr b1000 | Nr. b0 |
| --- | --- | --- | --- | --- | --- | --- | --- |
| 14 | 3 T Prisma Fit | 1.6 | 1.6 | 56 | 3900 | 30 | 10 |
| 9 | 3 T Prisma Fit | 1.6 | 1.6 | 80 | 4900 | 20 | 10 |
| 4 | 3 T Verio | 3.3 | 3.3 | 95 | 6600 | 30 | 5 |
| 3 | 3 T Verio | 3 | 3.3 | 95 | 5600 | 30 | 5 |
| 2 | 1.5 T Aera | 3.3 | 3.3 | 63 | 6400 | 24 | 4 |
| 1 | 1.5 T Aera | 3.3 | 3.3 | 71 | 5200 | 20 | 5 |
| 1 | 1.5 T Aera | 3.3 | 3.3 | 63 | 7000 | 24 | 4 |
| 1 | 3 T Prisma Fit | 1.6 | 1.6 | 59 | 4300 | 30 | 10 |
| 1 | 3 T Skyra | 1.8 | 1.8 | 80 | 5900 | 20 | 10 |
| 1 | 3 T Skyra | 3.3 | 3.3 | 63 | 8100 | 18 | 3 |
| 1 | 3 T Verio | 3.3 | 3.3 | 95 | 6810 | 30 | 5 |
| 1 | 3 T Verio | 3.3 | 3.3 | 95 | 6810 | 24 | 4 |
| 1 | 3 T Verio | 3.3 | 3.3 | 100 | 9500 | 20 | 1 |

N = number of patients with the respective scanning protocol parameters; TE = time to echo; TR = repetition time; Nr b1000. = number of b = 1000 s/mm^2^ diffusion gradient directions;; Nr b0 = number of images with b-value 0 (T2-weighted images).

**Image acquisition parameters as nuisance variables**

To control for a possible confounding effect of heterogeneous scanning protocols in the NBS analyses, we included four acquisition parameters as nuisance covariates:: interslice spacing, echo time, repetition time, and the number of b= 1000 s/mm2 diffusion gradient directions. These acquisition parameters showed high inter-dependence, as well as significant correlation with the clinical scores (see correlation matrix in Table S3). To reduce multicollinearity between the independent variables, we have aggregated the four acquisition parameters (after z-score normalization) into a single covariate using the principal component analysis. The scores derived from the loadings of the first component were then used as a single covariate. The summary of the principal component analysis is given in Table S4.

**Supplementary Table S3.** Correlation matrix between the acquisition parameters and clinical scores.

|  |  | DRS | CRS-R | inter-slice spacing | TE | TR |
| --- | --- | --- | --- | --- | --- | --- |
| DRS | Spearman’s rho | - |  |  |  |  |
|  | p-value | - |  |  |  |  |
|  |  |  |  |  |  |  |
| CRS-R | Spearman’s rho | - 0.80*** | - |  |  |  |
|  | p-value | <.001 | - |  |  |  |
|  |  |  |  |  |  |  |
| inter-slice spacing | Spearman’s rho | 0.01 | -0.06 | - |  |  |
|  | p-value | 0.97 | 0.69 | - |  |  |
|  |  |  |  |  |  |  |
| TE | Spearman’s rho | 0.04 | -0.12 | 0.66*** | - |  |
|  | p-value | 0.82 | 0.47 | <.001 | - |  |
|  |  |  |  |  |  |  |
| TR | Spearman’s rho | -0.06 | 0.014 | 0.87*** | 0.83*** | - |
|  | p-value | 0.69 | 0.93 | <.001 | <.001 | - |
|  |  |  |  |  |  |  |
| Nr b1000 | Spearman’s rho | 0.34* | -0.28 | -0.03 | -0.32* | -0.4* |
|  | p-value | 0.03 | 0.08 | 0.87 | 0.04 | 0.01 |

DRS = Disability Rating Score; CRS-R = Coma Recovery Scale – Revised; TE = time to echo; TR = repetition time; Nr b1000. = number of b = 1000 s/mm^2^ diffusion gradient directions; * p < .05, ** p < .01, *** p < .001

**Supplementary Table S4. Sumary of the principal component analysis**.

|  | | PC1 | PC2 | PC3 |
| --- | --- | --- | --- | --- |
| Explained variance ratio | | 0.62 | 0.25 | 0.10 |
| Feature loadings | |  |  |  |
|  | inter-slice spacing | 0.543 | -0.416 | 0.367 |
|  | TE | 0.534 | -0.004 | -0.845 |
|  | TR | 0.602 | 0.017 | 0.355 |
|  | Nr b1000 | -0.240 | -0.909 | -0.157 |
| Correlations with clinical scores | |  |  |  |
| DRS | Spearman’s rho | -0.05 | -0.34* | -0.15 |
|  | p-value | 0.77 | 0.03 | 0.35 |
| CRS-R | Spearman’s rho | -0.02 | 0.31 | 0.21 |
|  | p-value | 0.89 | 0.055 | 0.20 |

PC1 = first principal component; PC2 = second principal component; PC3 = third principal component; DRS = Disability Rating Score; CRS-R = Coma Recovery Scale – Revised; TE = time to echo; TR = repetition time; Nr b1000. = number of b = 1000 s/mm^2^ diffusion gradient directions; * p < .05, ** p < .01, *** p < .001

**REFERENCE**

1. Pozeg P, Jöhr J, Pincherle A, et al. Discriminating cognitive motor dissociation from disorders of consciousness using structural MRI. *NeuroImage Clin*. 2021;30:102651. doi:10.1016/j.nicl.2021.102651
